# The Late Miss Luckes

**Published:** 1919-02-22

**Authors:** E. W. Morris

**Affiliations:** the House Governor.


### THE LATE MISS LUCKES.

### THIRTY-NINE YEARS MATRON OF THE LONDON HOSPITAL.

**By Mr. E. W. Morris, the House Governor.**

I HAVE been asked to write down what I consider to be some of the chief characteristics of Miss Luckes, the late Matron of the London Hospital. If I had known Matron only for a few weeks or even months I think I should have had little difficulty in doing so, but as I have known and worked with her for more than twenty years, I find that I have been asked to perform a task quite beyond my powers. And that is because I should like you to understand something of Matron's character rather than her characteristics.

May I explain what I mean? If you ask a lad of ten what are the colours which are hidden in the rays of the white light of the sunshine, he will glibly tell you that if you throw a ray of white light through a prism, it will be broken up by the prism into its seven constituent colours—violet, indigo, blue, and the rest. That settles the matter with him, for he is only ten years old, and he goes on his way whistling. And he is quite right. He is speaking of characteristics. But if you were to put exactly the same question—what colours are hidden in the sunshine?—to a man who for twenty years had faced the stress and turmoil of life, I do not believe he would think about the prism at all. I think he would look back and remember the time when he had gazed on a sea of purple heather on Exmoor, or a field of flaming poppies in Norfolk, or a lake of blue-bells in a Kentish wood with the ghosts of last year's blue-bells shimmering above them, or the quiet sleepiness of yellows and browns of an English valley in Autumn. And he would be right too. All those colours are in the sunshine, and are part of it. But they are a measure of it as well as an analysis of it. I feel a little like that man as I look back these twenty years. To analyse Matron's characteristics is a small thing I wish I could make you see the measure of the work that Matron has done for us all.

### Miss Luckes Appointed Matron.

It is a long time ago, nearly forty years, since five ladies came before the House Committee in the old Committee Room, now the House Governor's office, as candidates for the post of matron to this hospital. The choice fell upon Miss Luckes, and I have sometimes wondered why. She was very young for such an important position—only twenty-four—and she had not had previous experience at any other large hospital. Although there are one or two members still attending Committee who were present on that day, I have never been able to satisfy my curiosity as to why they selected Miss Luckes. We are glad enough that they did.

### The Difficulties which had to be Faced.

We, who have been accustomed to the splendid organisation and high tone of the nursing and training at the "London," can hardly appreciate the outlook or the difficulties which had to be faced by the young matron when she took up her duties here. There was no training school of any sort. The women who acted as nurses were of the lowest type, and were engaged from outside. Even the sisters were advertised for. Those of us who cross the main hall about eleven at night may have seen a row of women waiting to be hired to do a night's scrubbing. Matron, many and many a time, has had to choose her night nurses from just such a row. They were treated with contempt by committee and staff. Most of them had to find their own food; all had to find part of it. Matron herself had to provide her own food when she came. Such a luxury as separate bedrooms was not thought of. There was one bath provided, and was considered sufficient for all the nurses, and there were about 130. Not a week but some of them were called before the committee for some offence, and the easy way they were treated by the committee shows how low their status was: a nurse, intoxicated on duty all night, was simply cautioned that it must not occur again; a nurse who had undoubtedly been cruel to a dying child was only told to be more kind in future; and I note the surprise of the committee when matron, just after her appointment, wished that a nurse should be dismissed who habitually entered up temperatures without taking them; matron was asked to reconsider the matter. I mention these things so that you may see what Miss Luckes had to face. There seemed to be no material on which she could make a beginning. And the worst of it all—the most damning to all progress—was that committee and staff, nurse and patient, were quite satisfied with things as they were; they wished for nothing better. "When there is no vision the people perish," and I take it that nursing was saved in that day in the London Hospital, and thereby saved in many another hospital, because there was one person who saw the vision and dreamed her dreams, and that one person was the young matron of twenty-four. Whence did she get her ideals? Who told her to look forward to and to work for all that you see around you to-day?

### True to Her Vision.

It pleases me to note that she was so true to her vision that within twenty-four hours of taking up her duties here she boldly faced the committee that had appointed her and told them plainly that the nursing staff at the hospital was inadequate both in quality and in numbers. That was a brave thing to do, and is the sort of thing that is done by those who see visions. And she had her immediate reward, for she succeeded in making the committee see that great things would be attained and that a better way lay ahead. On that day, and from that day, she fought inch by inch for her ideals. I think she saw on that day a great and noble army whose glory would be "self-sacrifice," great-hearted women coming from the north and south and east and west, pledged to give themselves and all their knowledge for the bettering of the sick, and especially the poor sick; she dreamed of a sympathy that was trained to be of greatest value, of kind-heartedness that was skilled, of true womanliness ennobled by orderliness and judgment—she saw the trained nurse. As I read these old minutes I can see her winning step by step towards the goal that was set before her.

### Better Conditions for the Nurses.

First, she fought for better conditions for the nurses who were here. She demanded that they should be fed, and fed properly, and one can see how slow the progress was when it is noted that even after the committee had agreed to give food, they did not think any refreshment was required between eight o'clock breakfast and five o'clock dinner, except beer and 4*d.* each to the sisters to buy bread and cheese. I wonder how many times

### THE LATE MISS LUCKES—(*continued*).

I have noted in the minutes in those first years, "Matron complained of the nurses' food"; "matron complained of the lack of variety in nurses' food, only one kind of pudding having been given for more than two years"; and so on, over and over agoin. And she fought so hard for better accommodation for nurses, for some little refinements in their dormitories. Her request for one extra bath is almost humorous; she says that the lack of this "luxury" seems to deter candidates of nice feeling from coming. "The better class of women make rather a point of conveniences for personal cleanliness," she pointed out. The committee had hardly yet come to regard a nurse as a "better class of woman," and Miss Luckes had much to teach them yet. Then she fought for better hours: it was not fair that nurses should have no definite hours off; it was not right that night nurses should be expected to "keep an eye on the ward" while the day staff all went to dinner; and point by point she won.

### Miss Luckes's Greatest Fight.

And then she began her greatest fight of all for the true *training* of nurses, the building-up of a skilled profession out of a crowd of undisciplined women. She mapped out what a course of training should be, as distinct from a time spent in wandering aimlessly about the wards; in the first year she was convinced that a year was not long enough to train a nurse—the then accepted period—and altered it to two, at which it has remained ever since. She began to give her own courses of lectures on nursing, and every year since then, as you know, she has never failed to give them. She persuaded the committee to ask two members of the staff to give a course of lectures every year—one on surgical and the other on medical nursing—and these lectures have never failed since she introduced them; she insisted that the sisters in the wards must themselves be the finest and most experienced of the nurses, and that they must be teachers themselves. And through it all we can see how matron herself was encouraging, and stimulating, and inspiring, making unkindness seem more unkind, slackness more contemptible, cheerfulness more worthy, meanness more mean, accuracy more desirable, womanliness more loveable. Through all the years she led; she saw the road farthest ahead; she made it to be counted a very fine thing to be a good nurse, the finest thing in the world to be a good woman. I need not write much more, but it may be of interest to know a few of the steps by which she, the nurses, and we all have attained, and so I jot them down as I find them recorded.

### System of Two Years' Training Begun.

She commenced the system of two years' training directly after she was appointed in 1880, and the first certificates of this hospital were issued in 1882. After long persuasion of the committee as to its advisability, she succeeded in starting the private staff, for nursing better-class patients who could pay, but who knew of no nursing but the Mrs. Gamp type, in 1886. In 1893 she won her battle as to the necessity of training nurses in the cookery of the sick room, and the sick-room cookery class was founded. It was held on four evenings a week. In 1893, too, she gained her point that it was to the committee's interest to encourage cleanliness among the nurses, and she then obtained the grant of 2s. 6d. a week towards washing expenses. Next year, 1894, Miss Luckes gained her point of the necessity of a medical examination for intending candidates, and Dr. Warner has carried out this duty at matron's request for twenty-five years.

### Examiners of Nurses Independent Outsiders.

She had always contended that it was advisable that the examiner to the nurses should be some one outside the hospital—a stranger to the nurses—and in 1894 she gained this point. This same year, 1894, Miss Luckes obtained the gratification of a long-felt wish that there should be some kind of institution attached to the hospital in which candidates could obtain a preliminary training before entering the hospital itself. Two houses were taken in Bow Road and were called Tredegar House. This was the forerunner of the magnificent training-school known to us all to-day in the handsome new building, the site of which was given by Lord Tredegar.

### The Nurses' Library.

Also in 1894 matron established the nurses' library. It had been an earnest wish of hers for many years. Next year, 1895, the sick-room for nurses was an accomplished fact; the provision of such accommodation had long been desired. Previously, of course, nurses were nursed, when ill, in the public wards with other patients.

### Miss Luckes's Study Classes for Probationers.

In 1896 she elaborated the present system of study classes for probationers, and in this year, too, great improvements were won for the nursing staff in the direction of holidays and time off. It is a comparatively small matter, but in the same year new beds were purchased throughout the hospital, and at matron's suggestion they were made of the present height instead of the low beds previously used. This was entirely to make it more convenient for nursing and to save the constant backache through having to stoop. The great and important step of making senior probationers take greater responsibility was commenced in 1897, when the silver "S" was introduced. The Garden of Eden was given by Mr. Vatcher to matron for the use of nurses, and nurses only, in 1898. The Marie Celeste Wards, in which nurses may obtain their midwifery training, a scheme for which matron had long worked, were opened in 1905. But I need say no more of all these steps forward on the initiative of Miss Luckes. They are all evidences of the burning energy within that foresaw all, planned all, fought, persuaded, appealed for all.

### A Splendid Character.

And all that I have reminded you of will help you to see for yourself the splendid character, uplifting, encouraging, ennobling, which has dwelt amongst us. At one time I have been amazed at her industry. No one worked so hard. Always up soon after six in the morning, never finished before next midnight. Her continuity of purpose was wonderful; her ideals held so bravely and so clearly fill us all with admiration; her unfailing cheerfulness and absolute refusal to be discouraged; her sympathy with all who were downhearted and downtrodden; her power of making prompt and sound decisions; her almost childlike delight in very simple things (how often she has told me of some dinner she has been to at an old friend's house, and her joy at "all the pretty dresses")—all these things are our own possession—she was all this for us. For to her "the London" and its reputation, its work, its movement, its good name, was everything. She belonged to "the London," she lived for "the London"; and now her great example lives in "the London," where, like old Matthew Audley, the first chaplain, she worked for forty years "carrying all kinds of consolation."

E. W. Morris.

